# A Culture-Free Lipidomics-Based Screening Test for Uropathogens

**DOI:** 10.1093/clinchem/hvaf164

**Published:** 2025-12-05

**Authors:** Linda K Nartey, Mert Pekcan, Jun Liu, Victor Yuen, Pamela Kibsey, Robert K Ernst, David R Goodlett, Michael X Chen

**Affiliations:** Department of Biochemistry and Microbiology, University of Victoria, Victoria, BC, Canada; Genome British Columbia Proteomics Centre, University of Victoria, Victoria, BC, Canada; Faculty of Veterinary Medicine, Department of Biochemistry, Ankara University, Ankara, Turkey; Department of Pathology and Laboratory Medicine, University of British Columbia, Vancouver, BC, Canada; Department of Pathology and Laboratory Medicine, University of British Columbia, Vancouver, BC, Canada; Department of Pathology, Laboratory Medicine & Medical Genetics, Vancouver Island Health Authority, Victoria, BC, Canada; Department of Pathology and Laboratory Medicine, University of British Columbia, Vancouver, BC, Canada; Department of Pathology, Laboratory Medicine & Medical Genetics, Vancouver Island Health Authority, Victoria, BC, Canada; Department of Microbial Pathogenesis, University of Maryland, Baltimore, MD, United States; Department of Biochemistry and Microbiology, University of Victoria, Victoria, BC, Canada; Genome British Columbia Proteomics Centre, University of Victoria, Victoria, BC, Canada; Department of Pathology and Laboratory Medicine, University of British Columbia, Vancouver, BC, Canada; Department of Pathology, Laboratory Medicine & Medical Genetics, Vancouver Island Health Authority, Victoria, BC, Canada; Division of Medical Sciences, University of Victoria, Victoria, BC, Canada

## Abstract

**Background:**

A rapid culture-free method is needed to improve diagnostic efficiency and guide timely antimicrobial therapy for urinary tract infections (UTIs). Previously, we utilized the lipidomics-based fast lipid analysis technique (FLAT) to screen for uropathogens by identifying distinctive microbial membrane lipid profiles, specifically lipid A in gram-negative and cardiolipin in gram-positive bacteria. This culture-free assay demonstrated high sensitivity (94%) in detecting gram-negative bacteria but poor sensitivity (51%) for gram-positive bacteria.

**Methods:**

In this study, we pretreated urine pellets with lysozyme prior to FLAT analysis to break down the peptidoglycan layer bacteria, thereby promoting the efficient release of cardiolipin. The limit of detection (LOD) for 4 gram-positive bacteria and *Escherichia coli* was evaluated using contrived samples with known CFU/mL values and varying concentrations of lysozyme. Subsequently, we validated the optimized method in a clinical cohort of 76 urine samples known to contain gram-positive bacteria as confirmed by urine culture.

**Results:**

Optimal sensitivity was achieved by treating 1 mL of urine pellets with 100 µg lysozyme and incubating for 60 minutes, resulting in a 100-fold increase in cardiolipin LOD and a 95% detection rate for gram-positive bacteria. Signal-to-noise ratio for lipid A was also improved. Polymicrobial urine cultures with gram-negative and gram-positive species were identified in 2 patients.

**Conclusions:**

The lysozyme-enhanced FLAT assay enables rapid and culture-free detection of both gram-negative and gram-positive uropathogens directly from urine. The unified workflow decreases the analytical turnaround time by at least 90% making it well-suited for high-throughput clinical laboratories.

## Introduction

Urinary tract infections (UTIs) are among the most common bacterial infections, accounting for millions of outpatient visits annually worldwide (1). Traditional urine culture methods, while considered the diagnostic gold standard, are time-consuming and resource intensive (2). UTIs are most frequently caused by *Escherichia coli*, accounting for nearly 80% of infections. The other 20% are composed mostly of *Klebsiella pneumonia*, *Proteus mirabilis*, and gram-positive bacteria such as *Enterococcus* spp., *Staphylococcus saprophyticus*, and *Streptococcus agalactiae* contributing significantly to disease burden (3–5). Rapid and accurate identification of uropathogens is essential for effective antimicrobial therapy, particularly in the context of rising antibiotic resistance.

In our previous study, we developed a mass spectrometry lipidomics-based culture-free workflow to directly detect microbial membrane lipids using lipid A and cardiolipin as biomarkers for gram-negative and gram-positive bacteria directly from specimen (3). The fast lipid analysis technique (FLAT) method used therein has a turnaround time (TAT) of <1 hour, to detect pathogens directly from specimen, which promises to eliminate the need for culture in the low-risk general population (6). We have previously reported that our lipid assay can efficiently detect several gram-negative bacteria such as *E. coli*, *Klebsiella*, *Proteus*, *Enterobacter*, and *Pseudomonas* spp. etc. (3, 6–8). While gram-negative bacteria were detected by FLAT at 94% sensitivity, this workflow was notably less effective at detecting gram-positive bacteria. Clinically significant gram-positive pathogens, such as *Enterococcus faecalis*, *Staphylococcus saprophyticus*, and *Streptococcus agalactiae* were inconsistently detected despite their known prevalence in UTIs. These gram-positive bacteria often contribute to complicated UTIs (9) and are particularly prevalent in high-risk populations that include patients with recurrent or complicated UTIs, patients with catheters or sexually transmitted infections, the elderly, and pregnant patients (10, 11).

Detecting gram-positive bacteria directly from urine using the FLAT lipidomics assay proved challenging, likely due to their thick peptidoglycan cell wall, which hinders the release of cardiolipin necessary for mass spectrometry detection. In contrast, gram-negative bacteria, which have a thin peptidoglycan layer, readily release lipid A. Given that we previously showed that sonication enhanced cardiolipin detection from gram-positive organisms, we hypothesized that enzymatic pretreatment before FLAT could offer similar benefits. Specifically, lysozyme hydrolyzes the β-1,4-glycosidic bonds between *N*-acetylmuramic acid and *N*-acetylglucosamine in the peptidoglycan layer of bacterial cell walls (12–14). By integrating lysozyme digestion before FLAT lipid extraction, we hypothesized that the yield of detectable gram-positive membrane lipids, specifically cardiolipin, would improve significantly. In this study, we first determined the optimal condition of lysozyme treatment and established the limit of detection (LOD) of our assay. Following these optimizations, we validated the method in a clinical cohort with confirmed gram-positive bacterial infections to assess detection efficiency.

## Materials and Methods

### Optimization of FLAT with Lysozyme Treatment

#### Limit of Detection Using Contrived Samples

This study was approved by the Vancouver Island Health Authority Research Ethics Board No. H21-037853785. Samples were anonymized discards from outpatients and inpatients. Clinical presentation was not available. Negative urine samples were obtained in sterile BD Vacutainer Urine C&S Preservative Tubes (BD) and allocated for FLAT analysis within 3 days of receipt. Samples were stored at 4°C until processed. To determine the LOD for each organism, contrived urine samples were prepared by spiking negative urine with known concentrations [colony-forming units (CFU)/mL] of 4 gram-positive and 1 gram-negative bacteria. Briefly, 1 mL aliquot of spiked urine samples were transferred into an Eppendorf tube and centrifuged at 8000*g* for 5 minutes to obtain bacterial pellets. Pellets were then treated with increasing amounts of lysozyme, specifically 10, 50, 100, 500, and 1000 µg at 20 K units/mg (MP Biomedicals) and incubated at 37°C for either 30 or 60 minutes.

All the samples were subsequently analyzed by FLAT. Briefly, 1 µL of lysozyme-treated urine pellet was spotted directly onto the matrix-assisted laser desorption/ionization (MALDI) target plate in triplicate. Next, 1 µL of a citric acid buffer containing 0.2 M anhydrous citric acid and 0.1 M trisodium citrate dihydrate (Fisher Chemical) was placed on top of each dried sample. The MALDI target plate was then heated at 100°C for 20 minutes in a humidified chamber. The MALDI plate was gently rinsed with sterile distilled water. Subsequently, 1 µL norharmane (10 mg/mL) matrix (Sigma Aldrich) dissolved in 2:1 vol/vol MS-grade chloroform and methanol (both from Fisher Chemical) was spotted onto the extracted lipid sample on a MALDI target plate, allowed to dry, and then analyzed in the negative ion mode on the trapped ion mobility spectrometry (TIMS) time of flight (TOF) flex MALDI-2 instrument (Bruker). For this study, stainless-steel, 96-well, MALDI target plates, specifically MFX μFocus plate with dimensions 12 × 8c 2600 µm 0.7 T (Hudson Surface Technology, Inc.) were used for FLAT analysis. Samples with no lysozyme treatment were used as a control. All samples were processed in triplicate across 3 independent days to ensure reproducibility. In this study, the LOD was defined as the lowest bacterial concentration (CFU/µL of urine) at which the diagnostic biomarker (lipid A for gram-negative, cardiolipin for gram-positive) could be reproducibly detected.

#### Urine Culture and Pathogen Identification by MALDI-TOF MS

The TimsTOF flex MALDI-2 instrument (Bruker) was used for all data collection. The TIMS was inactivated in this study. All analyses were conducted in negative ion mode. Analyses were conducted at 65% global intensity with 500 laser shots for each acquired mass spectrum. Mass spectra were collected between 1000 and 2400 *m/z*. Acquired mass spectral data were processed using the Bruker data analysis software (v.6.1) and using ESI-L low concentration tuning mix (Agilent Technologies) as a calibrant. Samples were called positive when diagnostic biomarker ions were detected with a signal-to-noise (S/N) ratio >10. Specifically, lipid A ions (*m/z* 1400–2400) were used to identify gram-negative bacteria, while cardiolipin species (*m/z* 1000–1400) were used to identify gram-positive bacteria. Following biomarker detection, the full lipid mass spectrum was compared against our microbial library (3) for species-level identification.

In routine standard of care, urine samples were plated on a blood and MacConkey agar and incubated for 18–24 hours to obtain colonies for bacterial identification. Individual colony was then spotted unto the MALDI plate for protein Biotyper (MBT Sirius; Bruker) identification using the 3.4.207.48./BDAL 11 organism library.

### Clinical Validation

**Fig. 1.**
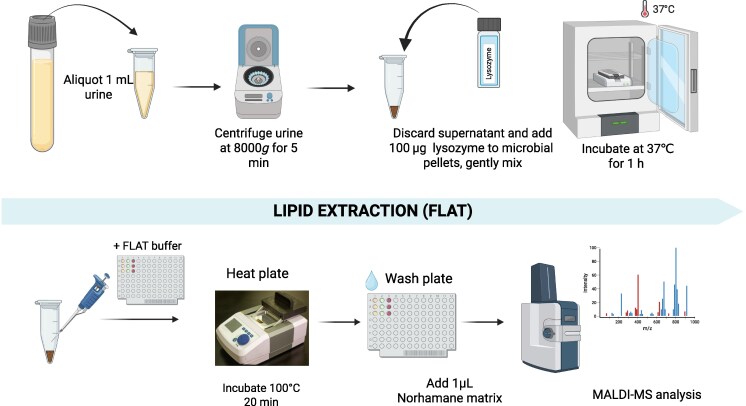
Experimental workflow for optimized direct-from-urine analysis of UTI samples by FLAT. The data analysis workflow for a sample involves a two stage determination of: the type of lipid(s) present, i.e., lipid A for gram-negative and cardiolipin for gram-positive organisms, and the organism from which this lipid(s) is likely derived. Both lipid type and species from which it is derived are achieved through spectral comparison to our in-house lipid reference library. Created in BioRender. Nartey, L. (2025) https://BioRender.com/5jrrcbc.

In this study, 76 gram-positive urine samples (Table 1) were collected from the microbiology laboratory of the Royal Jubilee Hospital, British Columbia, Canada. Most of the specimens were clean catch midstream urines collected from outpatients as part of routine diagnostic testing. Clinical symptoms were not provided on the requisition. All samples were anonymized clinical discards obtained from the microbiology laboratory. The requirement for consent was waived by local research ethics board. After routine culture workup was completed, aliquots were stored at 4°C and processed for FLAT analysis within 2–3 days of collection. Urine cultures confirmed the presence of gram-positive bacteria, and pathogen identification was carried out using the protein Biotyper. For FLAT analysis, 1 mL aliquot of each urine was transferred into an Eppendorf tube and treated with lysozyme at the optimal concentration previously determined from contrived LOD tests (described and illustrated in Fig. 1). All samples were processed in triplicate through the complete FLAT workflow to evaluate reproducibility.

**Table 1.** Patient demographics (*n* = 76).

	Females	Males
Adolescents (13–17 years)	0	1
Adults (18 years or older)	12	6
Older adults (>65 years)	26	31

#### Statistical Analysis

Mass spectral data were analyzed using Bruker data analysis (v.6.1) software, and pathogens were identified by comparing sample mass spectra to the previously developed microbial library. Sensitivity for correct pathogen identification was calculated using SE = TP/(TP + FN) × 100% where SE = sensitivity, TP = true positive, and FN = false negative.

## Results

### LOD Determination Using Contrived Samples

LOD studies were conducted using representative organisms: gram-positive species were *S. aureus*, *S. epidermidis*, *E. avium*, and *E. faecalis*, and the gram-negative species was *E. coli*. For the 4 gram-positive bacteria, FLAT identified bacteria at an average of 10^4^–10^5^ CFU/µL in samples without lysozyme treatment, using a S/N ratio of 10 for the base peak (Table 2, Supplemental Figs. 1A–5A). Incorporating lysozyme into the workflow improved detection limits to 102–103 CFU/µL (Table 2, Supplemental Figs. 1B–5B).

**Table 2.** LOD determination using contrived samples (CFU/μL).^a^

LOD without lysozyme treatment					
	Gram-positive				Gram-negative
Test strain	*S. aureus*	*S. epidermidis*	*E. avium*	*E. faecalis*	*E. coli*
CFU/µL	10^4^	10^5^	10^4^	10^5^	10^2^

LOD with lysozyme treatment										
	Gram-positive								Gram-negative *E. coli*	
Test strain	*S. aureus*		*S. epidermidis*		*E. avium*		*E. faecalis*			
Incubation (mins)	30	60	30	60	30	60	30	60	30	60
Amount of lysozyme (µg/mL)										
10 (CFU/µL)	10^4^	10^4^	10^4^	10^4^	10^4^	10^4^	10^5^	10^4^	10^2^	10^2^
50 (CFU/µL)	10^4^	10^4^	10^4^	10^4^	10^4^	10^4^	10^4^	10^4^	10^2^	10^2^
100^b^ (CFU/µL)	10^2^	10^2^	10^3^	10^3^	10^3^	10^2^	10^3^	10^3^	10^2^	10^2^
500 (CFU/µL)	10^2^	10^2^	10^3^	10^3^	10^3^	10^2^	10^3^	10^3^	10^2^	10^2^
1000 (CFU/µL)	10^2^	10^2^	10^3^	10^3^	10^2^	10^2^	10^3^	10^3^	10^2^	10^2^

^a^Bacterial counts are expressed as CFU/µL, based on a 1 mL bacterial stock with 1 µL spotted per test.

^b^Indicates optimal conditions.

The combination of 100 µg lysozyme per 1 mL urine pellet and a 60-minute incubation provided the most efficient detection rates. Lower lysozyme concentrations and shorter incubation times did not significantly enhance detection, suggesting incomplete lysis. Conversely, higher lysozyme concentrations (500 and 1000 µg per 1 mL pellet) showed no improvement. Table 2 confirmed that lysozyme treatment did not affect the LOD for gram-negative bacteria such as *E.* coli; however, it did lead to enhanced signal/noise for lipid A. Based on these findings, we determined that treating each 1-mL urine pellet with 100 µg of lysozyme and incubating for 60 minutes provided optimal conditions for efficient cardiolipin detection.

### Clinical Validation of Improved Detection of Uropathogens with Optimized FLAT

**Fig. 2.**
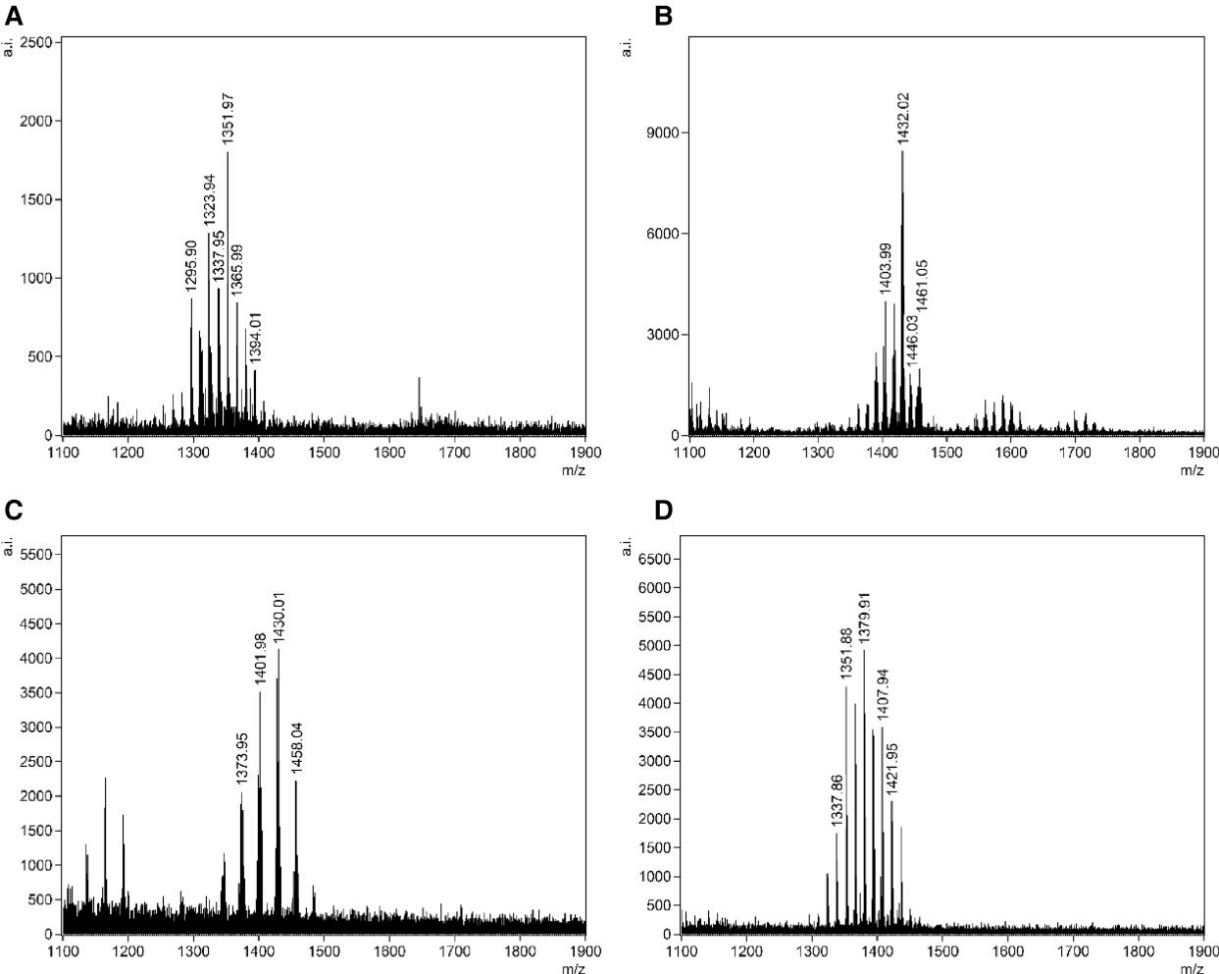
Representative FLAT mass spectra of detected gram-positive uropathogens. (A) *S. aureus*, (B) *E. faecalis*, (C) *A. urinae*, and (D) *S. epidermidis*. The *x*-axis shows the *m/z*, and the *y*-axis indicates the relative ion intensity (arbitrary units). Peaks corresponding to cardiolipin species are labeled with their respective *m/z* values.

**Fig. 3.**
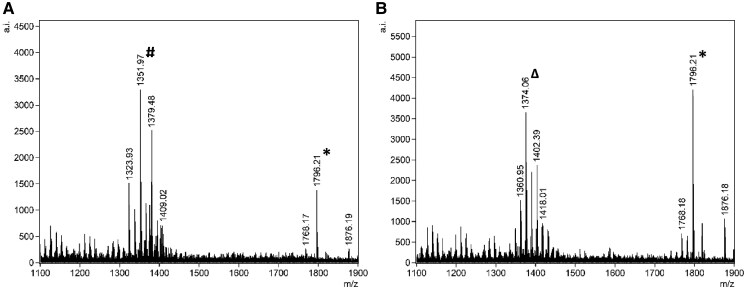
Representative FLAT mass spectra of detected polymicrobial samples. (A) *S. aureus*^#^ and E. coli* and (B) *A. urinae*^Δ^ and E. coli*. The *x*-axis shows the *m/z*, and the *y*-axis indicates the relative ion intensity (arbitrary units). Peaks corresponding to cardiolipin and lipid A are labeled with their respective *m/z* values.

Following optimization, we transitioned from testing contrived samples to analyzing clinical urine specimens obtained from patients. The optimized FLAT method incorporating lysozyme treatment significantly enhanced the detection of gram-positive uropathogens (Fig. 2, Supplemental Fig. 6) including *S. aureus*, *E. faecalis*, *A. urinae*, and *S. epidermidis*, and also enabled the detection of 2 polymicrobial samples (Fig. 3). Most samples contained single organisms. Two samples were polymicrobial [gram-positive plus gram-negative (*E. coli*)], and both were correctly identified by FLAT. A total of 76 known gram-positive urines containing 13 unique pathogens confirmed by culture were included. FLAT was able to correctly identify all 13 unique pathogens in 72 samples demonstrating a 95% agreement (Table 3). Four undetected samples (FNs) included *E. faecalis*, *Alloscardovia omnicolens*, *Staphylococcus haemolyticus*, and *S. lugdunensis*. The spectra from these 4 false positive samples confirmed the absence of detectable cardiolipin peaks (*m/z* 1300–1400). No signals corresponding to cardiolipin were observed above or below the S/N threshold, indicating true negatives rather than weak or misidentified detections. Of the 76 urine samples analyzed, 69 (91%) yielded consistent results across all 3 replicates, while 7 showed partial agreement. Samples were considered positive when at least 2 replicates produced matching spectral profiles (Supplemental Table 1).

**Table 3.** Detection of known gram-positive uropathogens by FLAT with lysozyme treatment.^a^

Known gram-positive	ID by culture	ID by FLAT
*Aerococcus sanguinicola*	2	2
*Aerococcus urinae* ^b^	6	6
*Enterococcus faecium*	6	6
*Staphylococcus aureus* ^c^	10	10
*Staphylococcus cohnii*	2	2
*Staphylococcus epidermidis*	6	6
*Staphylococcus saprophyticus*	1	1
*Streptococcus agalactiae* Group B	13	13
*Streptococcus pyogenes*	1	1
*Alloscardovia omnicolens*	**3**	**2**
*Enterococcus faecalis*	**21**	**20**
*Staphylococcus haemolyticus*	**2**	**1**
*Staphylococcus lugdunensis*	**3**	2
**Total**	**76**	**72 (95*%)***

^a^ID by culture: protein Biotyper and the 3.4.207.48./BDAL 11 organism library.

^b,c^Both polymicrobial with *E. coli.*

## Discussion

Urine culture is currently considered the gold standard for UTI diagnosis, but its main drawback is the lengthy TAT of 24–48 hours. Developing rapid and affordable diagnostic tools is essential to increase efficiency and reduce the use of empiric antimicrobial therapy.

In this study, we build on our previous work introducing the FLAT assay, a rapid, lipid-based method for UTI diagnosis that enables direct lipid extraction on a MALDI plate and pathogen detection within 1 hour of sample collection (3). While the FLAT assay detected gram-negative bacteria from clinical urine samples with 94% sensitivity, it was notably less effective at identifying gram-positive pathogens, which is a significant limitation for UTI diagnostics. Sonication of gram-positive-containing samples prior to FLAT analysis improved detection rates from 8% to 51%, suggesting that membrane disruption could enhance sensitivity. However, sonication poses risks of generating infectious aerosols, making it unsuitable for routine clinical use. As an alternative, we explored enzymatic cell wall disruption by adding lysozyme to urine samples before the FLAT assay to determine if it could improve the detection of gram-positive bacteria in UTI diagnostics.

Lysozyme is widely recognized for its capacity to degrade gram-positive bacterial cell walls (12, 13, 15), although its efficacy can differ among bacterial species and under varying enzymatic conditions. To leverage this property, we employed lysozyme pretreatment to break down peptidoglycan and weaken cell walls, thereby promoting the efficient release of cardiolipin during the FLAT lipid extraction process. The addition of lysozyme notably enhanced the detection of cardiolipin, reducing the LOD of cardiolipin for gram-positive bacteria by 100-fold to ≤10^3^ CFU/µL. The LOD of lipid A from gram-negative bacteria remained outstanding (10^2^ CFU/µL) with improved S/N ratio. The study satisfied the pre-established pass criterion requiring a S/N ratio exceeding 10. This ratio exceeds the performance expectation for LOD (S/N > 3) set by clinical best practice guideline (16).

The optimized FLAT protocol, which consisted of the addition of 100 µg lysozyme per 1 mL urine pellet followed by a 60-minute incubation, resulted in a total TAT of approximately 2 hours, offering a rapid and unified protocol for the simultaneous detection of both gram-negative and gram-positive pathogens.

During clinical validation, the optimized FLAT protocol increased the detection rate of gram-positive bacteria from 51% to 95%. In this clinical cohort (*n* = 76), 15 unique gram-positive species were present and identified by FLAT. Enterococcus species were the most common (35%) uropathogen. The optimized FLAT assay demonstrated 100% agreement with urine culture for 11 out of 15 uropathogens. The 4 undetected samples (FNs) included *E. faecalis* (not vancomycin-resistant *Enterococcus*), *A. omnicolens*, *S. haemolyticus*, and *S. lugdunensis*. After review of these cases with the hospital’s medical microbiologists, no pre- nor postanalytical issues were identified. The 4 negative urine samples took the usual 18–24 hours to grow with reported colony counts of >10^4^ CFU/mL. Our assay was unable to identify pathogens in these samples despite presumably adequate bacterial load. We suspect that differences in peptidoglycan composition and structure may have various degree of resistance to lysozyme activity. Ragland and Criss previously reported that differences in peptidoglycan composition among bacterial species may affect lysozyme-mediated lysis efficiency (17). Pathogens with a thicker peptidoglycan layer may require higher lysozyme concentrations to release cardiolipin effectively. Within the same bacterial species, different strains can exhibit similar variations. While the core structure is retained, variations in secondary modifications can markedly affect lysozyme lysis efficiency (18, 19). Such occurrences were infrequent (5%) in our cohort since the lysozyme concentration used was effective for most samples. However, the availability of samples was insufficient to explore the effects of higher lysozyme concentrations.

Polymicrobial growth in urine is a common finding in catheter-associated infections, immunocompromised patients, and cases of recurrent or complicated UTIs (20). Previously, we demonstrated that FLAT can independently identify multiple mixed species when spiked into the same urine sample (3). In this study, the optimized FLAT assay was able to correctly identify 2 urine samples containing both gram-negative and gram-positive species. One sample contained *S. aureus* and *E. coli*, and the other contained *A. urinae* and *E. coli*. These findings demonstrate that lysozyme pretreatment significantly improves the detection of gram-positive pathogens while maintaining the ability of the assay to identify gram-negative bacteria, allowing for comprehensive pathogen profiling from a single urine specimen.

Additional research is warranted to validate the performance of the optimized FLAT assay in special populations, such as individuals with compromised urinary tract anatomy and impaired immune systems. This may challenge the ability of the FLAT assay to detect microbes direct from specimen in atypical samples and those containing rare pathogens. The use of heat-resistant lysozyme variants could also be explored to further reduce TAT to 1 hour by conducting the lysozyme digestion during the 1-hour standard FLAT incubation. Although *S. aureus* is reported to be inherently resistant to lysozyme-mediated lysis (21), our pilot testing showed that, under FLAT assay conditions, lysostaphin did not provide additional benefit over lysozyme for *S. aureus* detection. This may be because other steps in our protocol including heating samples at 100°C for 20 minutes in a humidified chamber and/or the use of high concentrations lysozyme, also contributed to *S. aureus* lysis. Overall, lysozyme treatment, combined with our sample processing, was sufficient for detecting *S. aureus* and other uropathogens. Beyond UTIs, this approach holds promise for direct pathogen detection in other clinical specimens, such as blood culture bottles, cerebrospinal fluid, and bronchoalveolar lavage fluid, potentially expanding its utility to bloodstream and other infections.

## Conclusion

This study highlights the effectiveness of lysozyme treatment in improving the direct detection of gram-positive uropathogens from urine samples by FLAT. The optimized FLAT method achieved an overall detection rate of 95% in a clinical cohort of 76 patients with confirmed gram-positive infections. Importantly, combining lysozyme with a 60-minute incubation at 37°C significantly enhanced the release and detection of cardiolipins from gram-positive bacteria, while maintaining reliable detection of lipid A from gram-negative species; thereby enabling a unified workflow for comprehensive screening of UTIs. Moreover, the optimized FLAT method successfully identified polymicrobial samples containing gram-positive and gram-negative bacteria. This streamlined workflow will decrease the analytical TAT by at least 90% making it well-suited for high-throughput clinical laboratories.

## Supplemental Material

Supplemental material is available at *Clinical Chemistry* online.

## Supplementary Material

hvaf164_Supplementary_Data

## Nonstandard Abbreviations

UTI, urinary tract infection; FLAT, fast lipid analysis technique; LOD, limit of detection; TAT, Turnaround Time; CFU, colony-forming units; TIMS, trapped ion mobility spectrometry; S/N, signal-to-noise (ratio); FN, false negative.

## References

[1] Díaz JM, Dozois CM, Avelar-González FJ, Hernández-Cuellar E, Pokharel P, de Santiago AS, et al The Vacuolating Autotransporter Toxin (Vat) of *Escherichia coli* causes cell cytoskeleton changes and produces non-lysosomal vacuole formation in bladder epithelial cells. Front Cell Infect Microbiol 2020;10:299. 32670893 10.3389/fcimb.2020.00299 PMC7332727

[2] Flores-Mireles AL, Walker JN, Caparon M, Hultgren SJ. Urinary tract infections: epidemiology, mechanisms of infection and treatment options. Nat Rev Microbiol 2015;13:269–84. 25853778 10.1038/nrmicro3432 PMC4457377

[3] Nartey LK, Mikhael A, Pětrošová H, Yuen V, Kibsey P, Pekcan M, et al A lipidomics-based method to eliminate negative urine culture in general population. J Clin Microbiol 2024;62:e0081924. 39283074 10.1128/jcm.00819-24 PMC11481538

[4] Mandracchia VJ, Hayes DW, Yoho RM, Diagnosis HM. Differential and treatment options. Nat Rev Microbiol 2000;13:269–84.

[5] Codelia-Anjum A, Lerner LB, Elterman D, Zorn KC, Bhojani N, Chughtai B. *Enterococcal* urinary tract infections: a review of the pathogenicity, epidemiology, and treatment. Antibiotics 2023;12:778. 37107140 10.3390/antibiotics12040778 PMC10135011

[6] Sorensen M, Chandler CE, Gardner FM, Ramadan S, Khot PD, Leung LM, et al Rapid microbial identification and colistin resistance detection via MALDI-TOF MS using a novel on-target extraction of membrane lipids. Sci Rep 2020;10:21536. 33299017 10.1038/s41598-020-78401-3 PMC7725828

[7] Leung LM, Fondrie WE, Doi Y, Johnson JK, Strickland DK, Ernst RK, et al Identification of the ESKAPE pathogens by mass spectrometric analysis of microbial membrane glycolipids. Sci Rep 2017;7:6403. 28743946 10.1038/s41598-017-04793-4 PMC5526941

[8] Fondrie WE, Liang T, Oyler BL, Leung LM, Ernst RK, Strickland DK, et al Pathogen identification direct from polymicrobial specimens using membrane glycolipids. Sci Rep 2018;8:15857. 30367087 10.1038/s41598-018-33681-8 PMC6203844

[9] Kline KA, Lewis AL. Gram-positive uropathogens, polymicrobial urinary tract infection, and the emerging microbiota of the urinary tract. In: Mulvey MA, Klumpp DJ, Stapleton AE, editors. Urinary tract infections: molecular pathogenesis and clinical management. 2nd Ed. Washington (DC): ASM Press; 2016. p. 459-502. 10.1128/microbiolspec.UTI-0012-2012 PMC4888879 27227294

[10] Morgan JA, Zafar N, Cooper DB. Group B Streptococcus and pregnancy. Treasure Island (FL): StatPearls; 2024. 29494050

[11] Sizar O, Leslie SW, Unakal CG. Gram-positive bacteria. Treasure Island (FL): StatPearls; 2023. 29261915

[12] Ferraboschi P, Ciceri S, Grisenti P. Applications of lysozyme, an innate immune defense factor, as an alternative antibiotic. Antibiotics 2021;10:1534. 34943746 10.3390/antibiotics10121534 PMC8698798

[13] Primo ED, Otero LH, Ruiz F, Klinke S, Giordano W. The disruptive effect of lysozyme on the bacterial cell wall explored by an in-silico structural outlook. Biochem Mol Biol Educ 2018;46:83–90. 29131507 10.1002/bmb.21092

[14] Schleifer KH, Kandler O. Peptidoglycan types of bacterial cell walls and their taxonomic implications. Bacteriol Rev 1972;36:407–77. 4568761 10.1128/br.36.4.407-477.1972 PMC408328

[15] Ibrahim H, Aoki T, Pellegrini A. Strategies for new antimicrobial proteins and peptides: lysozyme and aprotinin as model molecules. Curr Pharm Des 2005;8:671–93. 10.2174/1381612023395349 11945164

[16] Clarke W, Molinaro JR, Bachmann ML, Botelho CJ, Cao Z, French D, et al CLSI C62 Liquid chromatography-mass spectrometry methods. 2nd Ed. Wayne (PA): Clinical and Laboratory Standards Institute (CLSI); 2022.

[17] Ragland SA, Criss AK. From bacterial killing to immune modulation: recent insights into the functions of lysozyme. PLoS Pathog 2017;13:e1006512. 28934357 10.1371/journal.ppat.1006512 PMC5608400

[18] Vollmer W, Blanot D, De Pedro MA. Peptidoglycan structure and architecture. FEMS Microbiol Rev 2008;32:149–67. 18194336 10.1111/j.1574-6976.2007.00094.x

[19] Garde S, Chodisetti PK, Reddy M. Peptidoglycan: structure, synthesis, and regulation. EcoSal Plus 2021;9:eESP-0010-2020. 10.1128/ecosalplus.ESP-0010-2020 PMC11168573 33470191

[20] Nicolle LE . Infections associated with urinary catheters. In: Schlossberg D, editor. Clinical infectious disease. 2nd Ed. Cambridge (United Kingdom): Cambridge University Press; 2015. p. 722–7.

[21] Bera A, Biswas R, Herbert S, Kulauzovic E, Weidenmaier C, Peschel A, et al Influence of wall teichoic acid on lysozyme resistance in *Staphylococcus aureus*. J Bacteriol 2007;189:280–3. 17085565 10.1128/JB.01221-06 PMC1797201

