# Supplementary material for: A Culture-Free Lipidomics-Based Screening Test for Uropathogens
Source: Clin Chem. 2025 Dec 5;72(3):381–9. doi: 10.1093/clinchem/hvaf164 (PMC13017057; doi:10.1093/clinchem/hvaf164)
Supplement: hvaf164_Supplementary_Data [file hvaf164_supplementary_data.pdf]

SUPPLEMENTAL INFORMATION

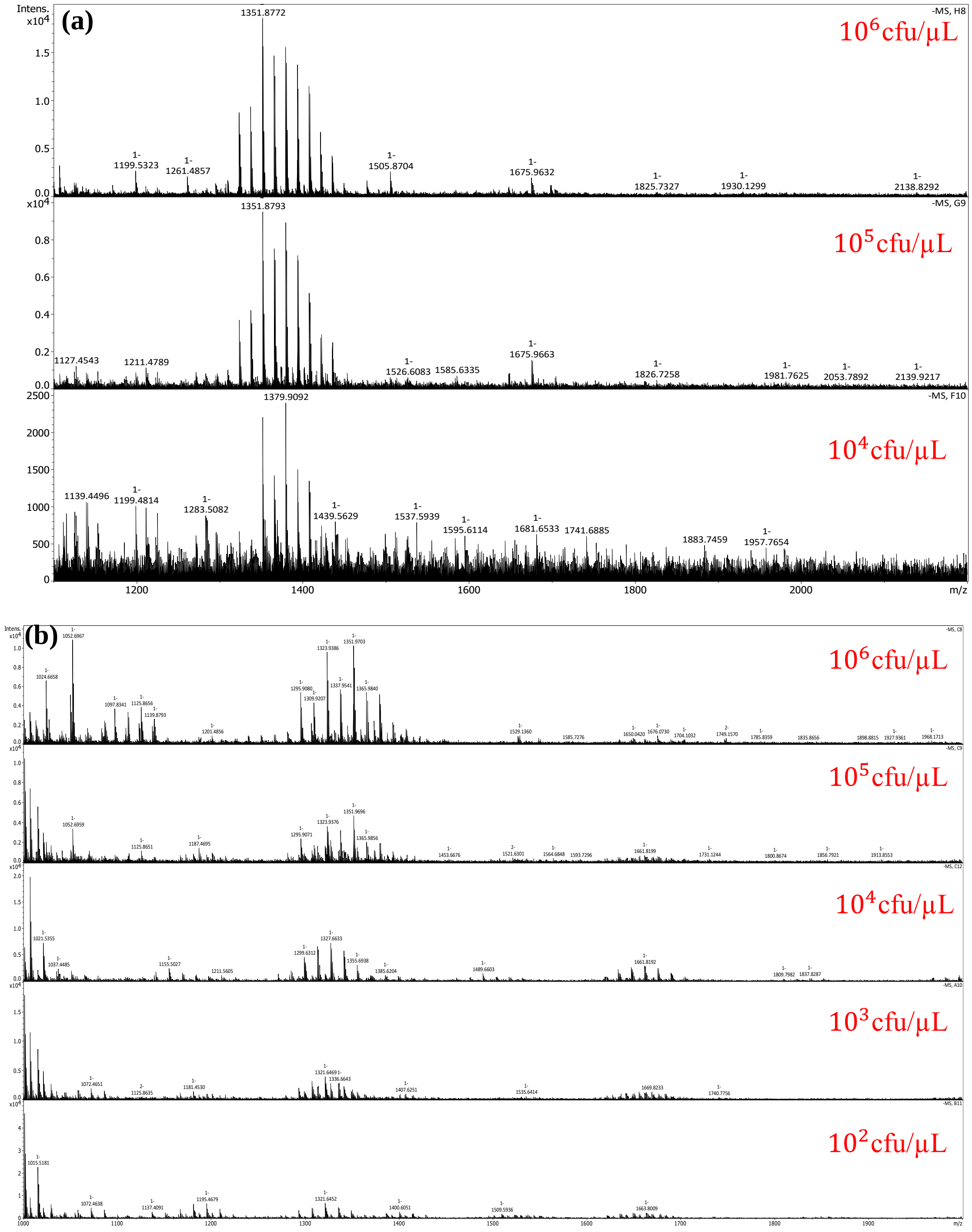

Figure 1. LOD determination of *S. aureus* (a) Urine-spiked S. aureus without Lysozyme treatment (b) Urine-spiked *S. aureus* with Lysozyme treatment. All samples were treated with 0.1mg lysozyme and incubated for 1hr

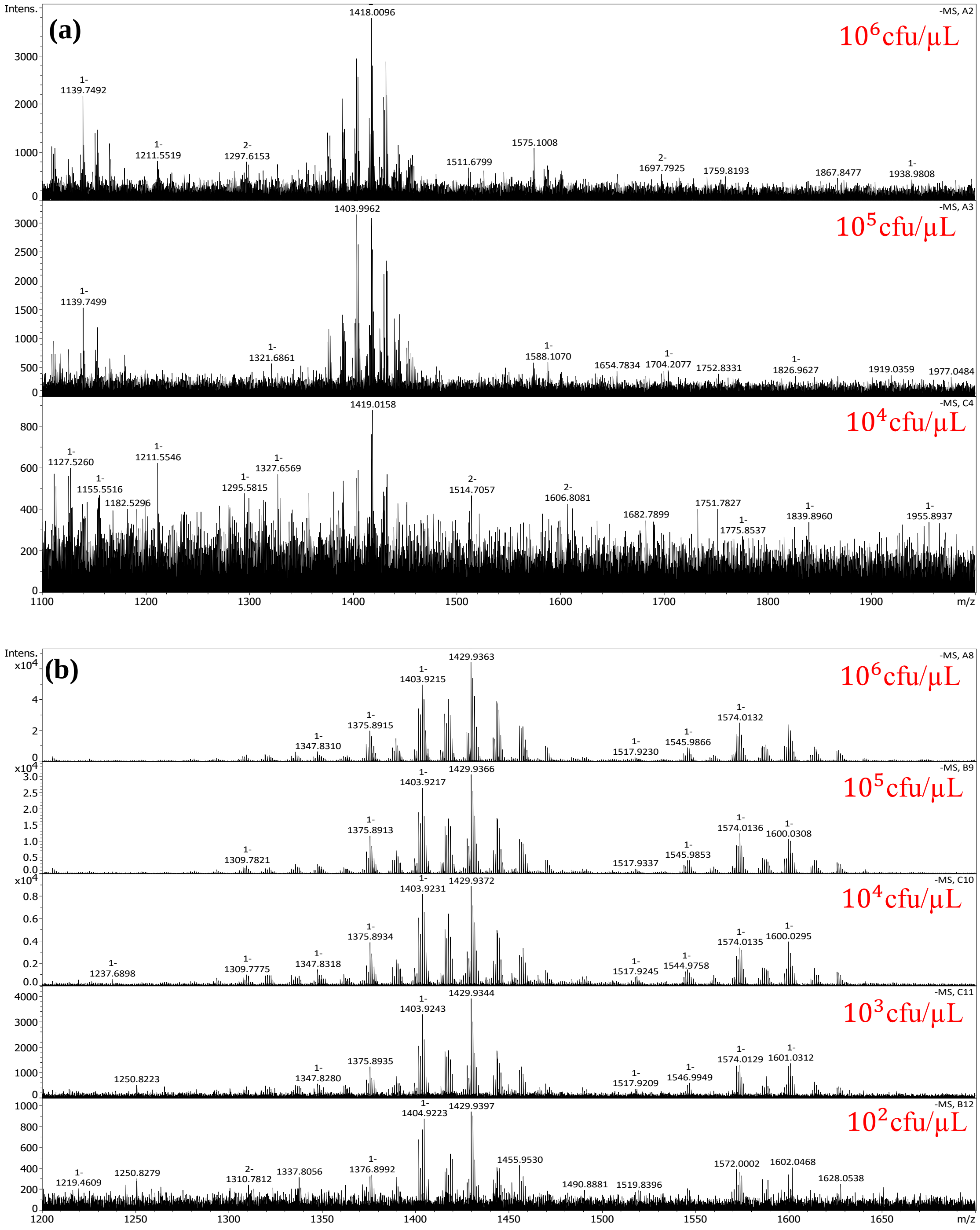

Figure 2. LOD determination of *E. avium* (a) Urine-spiked *E. avium* without Lysozyme treatment (b) Urine-spiked *E. avium* with Lysozyme treatment. All samples were treated with 0.1mg lysozyme and incubated for 1hr.

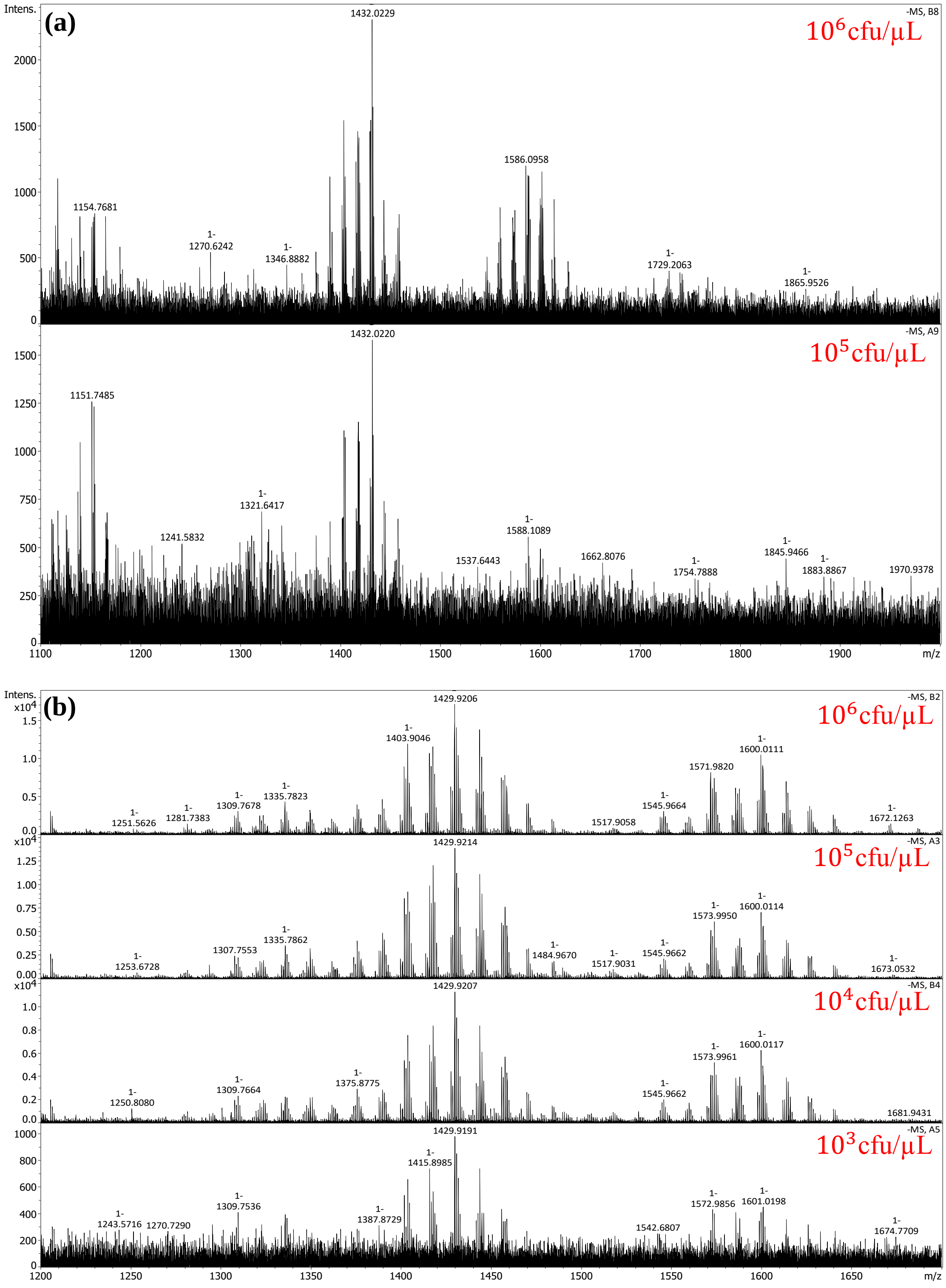

Figure 3. LOD determination of *E. faecalis* (a) Urine-spiked *E. faecalis* without Lysozyme treatment (b) Urine-spiked *E. faecalis* with Lysozyme treatment. All samples were treated with 0.1mg lysozyme and incubated for 1hr.

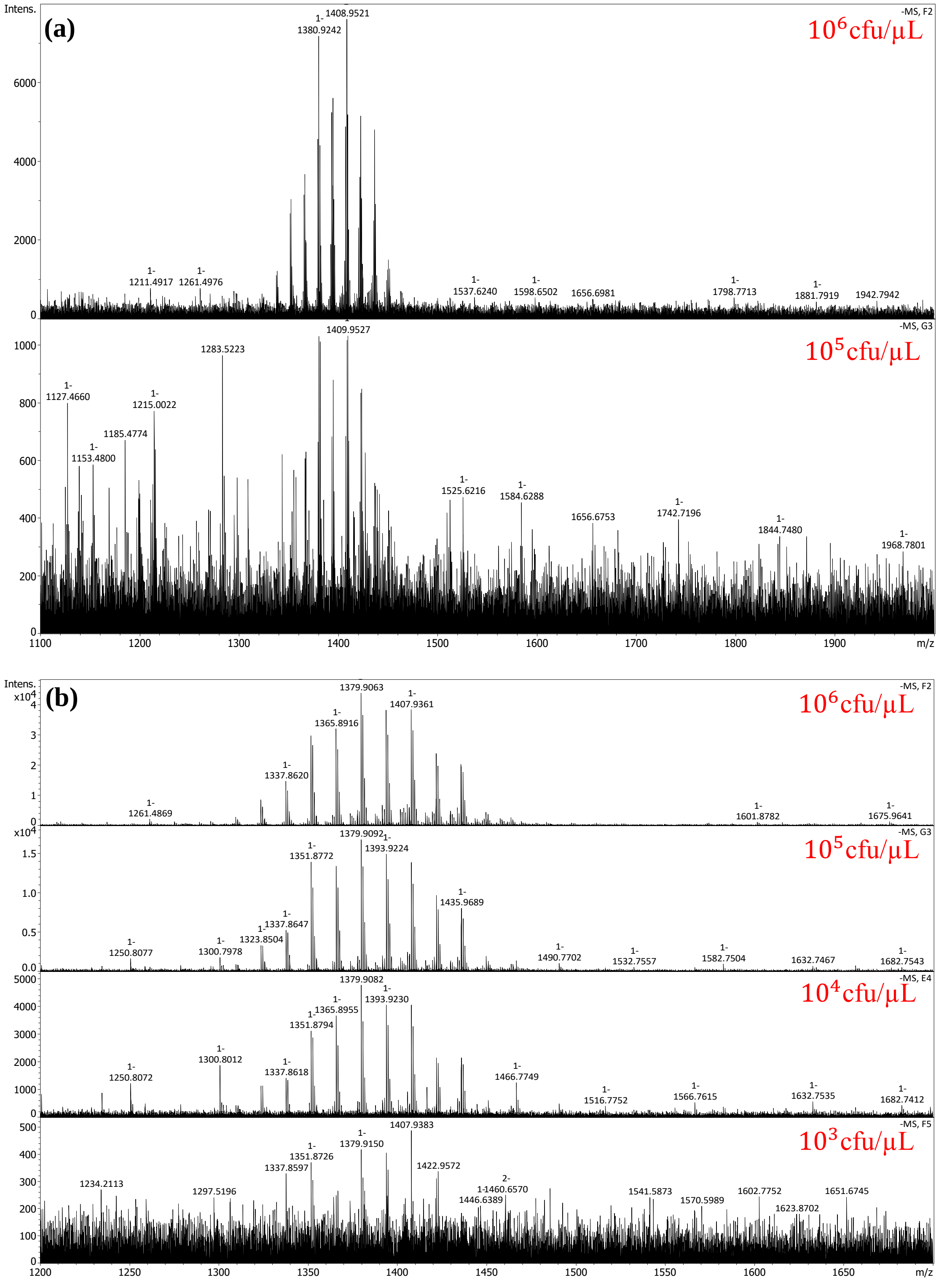

Figure 4.LOD determination of *S. epidermidis* (a) Urine-spiked *S. epidermidis* without Lysozyme treatment (b) Urine-spiked *S. epidermidis* with Lysozyme treatment. All samples were treated with 0.1mg lysozyme and incubated for 1hr.

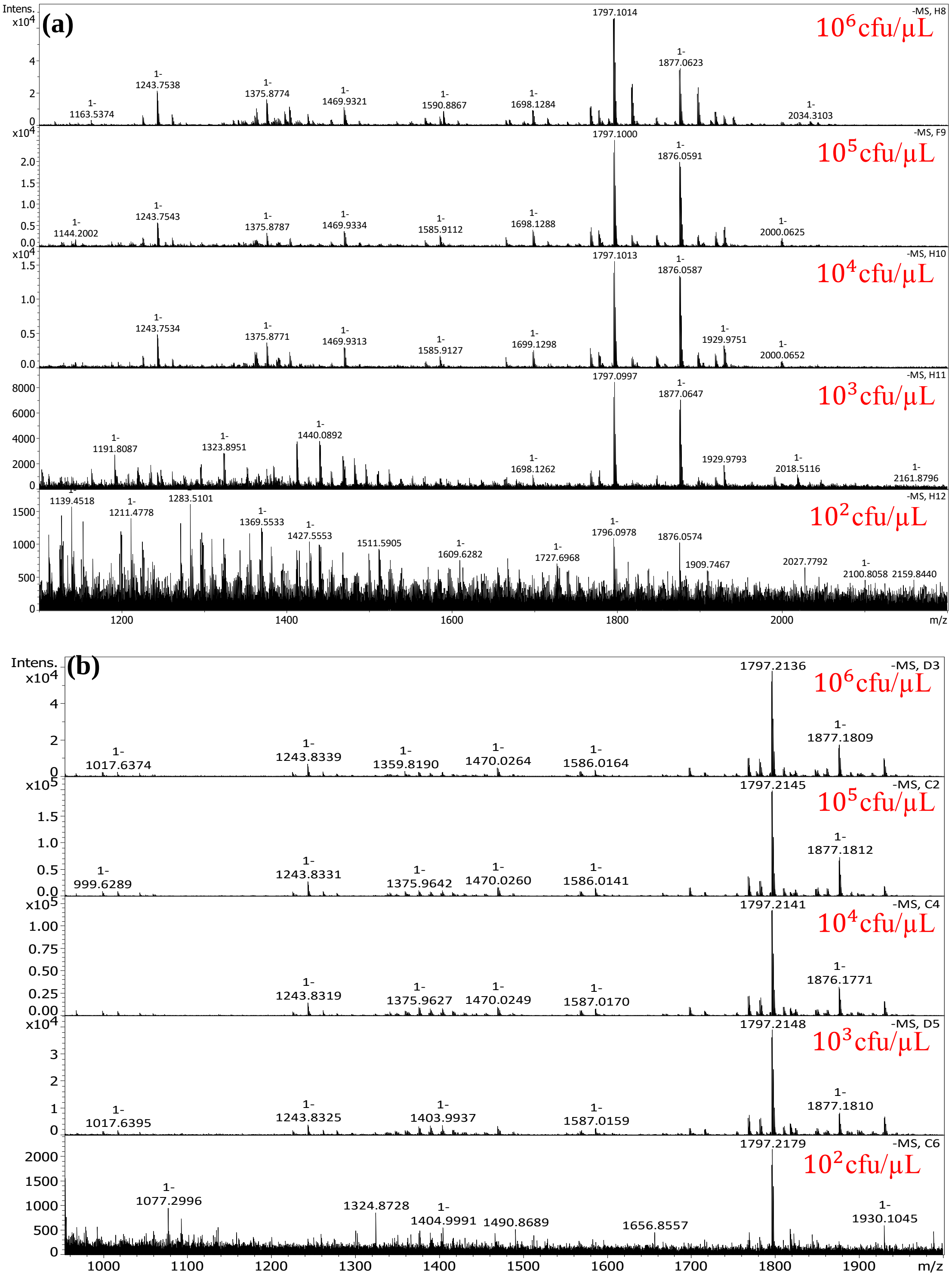

Figure 5. LOD determination of *E. coli* (a) Urine-spiked *E. coli* without Lysozyme treatment (b) Urine-spiked *E. coli* with Lysozyme treatment. All samples were treated with 0.1mg lysozyme and incubated for 1hr.

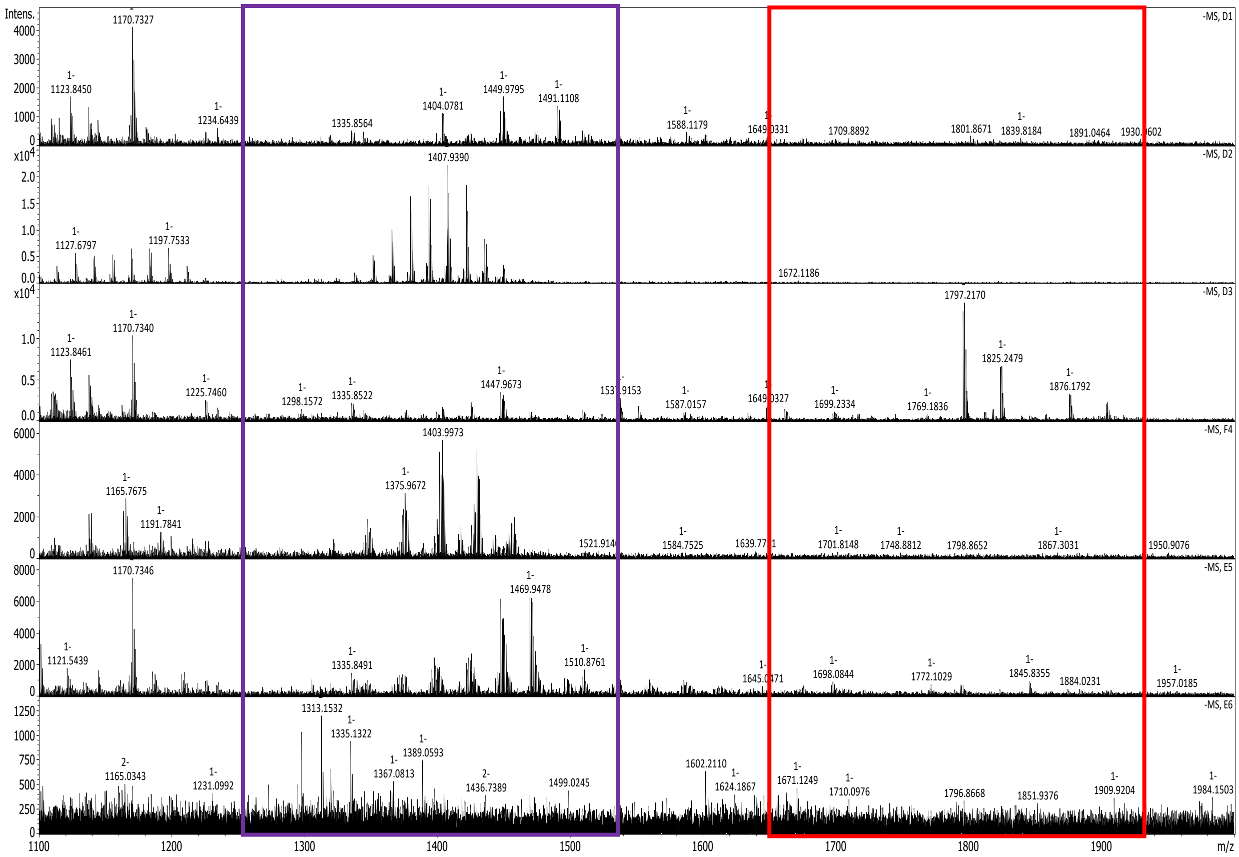

Figure 6. Representative raw spectra of 6 randomly selected samples showing detected cardiolipin (purple box) and lipid A (red box) directly from patient urine via FLAT after Lysozyme treatment.

Of the 76 urine samples analyzed in this study, 69 (91%) produced consistent results across all three replicates. Seven samples showed partial consistency, with two of the three replicates testing positive and displaying the same spectral patterns. A sample was classified as positive when at least two of three replicates yielded concordant results. The inconsistent samples are summarized in the table below.

Table 1. Summary of urine specimens with partial concordance across triplicate analyses.

SAMPLE ID	MICROBE	REPRODUCIBILITY BETWEEN REPLICATES
37652	*Enterococcus faecalis*	2 / 3
37663	*Streptococcus agalactiae*	2 / 3
37685	*Enterococcus faecalis*	2 / 3
37689	*Enterococcus faecium*	2 / 3
37698	*Staphylococcus epidermidis*	2 / 3
37700	*Streptococcus agalactiae*	2 / 3
37707	*Aerococcus urinae*	2 / 3
